# The Effect of Simvastatin on Glucose Homeostasis in Streptozotocin Induced Type 2 Diabetic Rats

**DOI:** 10.1155/2013/274986

**Published:** 2013-02-25

**Authors:** Lulu Wang, Guanglan Duan, Yong Lu, Shuguang Pang, Xianping Huang, Qiang Jiang, Ningning Dang

**Affiliations:** ^1^School of Medicine, Shandong University, Jinan, Shandong 250013, China; ^2^Department of Medicine, Jinan Central Hospital Shandong University, Jinan, Shandong 250013, China

## Abstract

*Objective*. To investigate the effect of simvastatin on glucose homeostasis in streptozotocin induced type 2 diabetic rats. *Methods*. Forty male Wistar rats were randomly divided into four groups. Normal control rats were fed with standard diet, others were fed with high-fat diet. Diabetic rats were induced by a single intraperitoneal injection of STZ. The simvastatin intervention rats were fed with simvastatin during the experiment process, and the simvastatin treatment rats were fed with simvastatin after diabetes rats were induced. We measured body weight, fasting plasma glucose, cholesterol, high-density lipoprotein cholesterol, and triglyceride after an overnight fast. *Results*. The FPG was higher in diabetic rats when compared to normal control ones; the simvastatin intervention rats had a higher FPG compared to the diabetic rats and were more easily be induced to diabetes at the end of 4 weeks, FPG level of simvastatin treatment rats was increased compared with diabetic model rats after 12 weeks. *Conclusion*. These data indicate that simvastatin intervention rats may cause hyperglycemia by impairing the function of islet **β** cells and have an adverse effect on glucose homeostasis, especially on FPG level.

## 1. Introduction

Type 2 diabetes mellitus is ordinarily associated with dyslipidemia, which presents a synergistic risk factor for cardiovascular disease [1]. Statins, the 3-hydroxy-3-methylglutaryl-coenzyme A (HMG-CoA) reductase inhibitors, are inhibitors of cholesterol synthesis. Statin therapy is effective for reduction of cardiovascular events [2] and is commonly recognized as being safe and well tolerated [3]. Furthermore, statin therapy has a close relationship with different doses of statin in cardiovascular patients. Intensive-dose statin therapy provides a significant benefit for preventing cardiovascular events compared with moderate-dose statin therapy [4].

 More recently, some studies [5] have reported that statin therapy may affect glucose homoeostasis in type 2 diabetes patients. However, the potential of statin use to affect glucose metabolism has been controversial [6]. Some studies provided that statins therapy have been linked to increased risk of developing diabetes mellitus [7, 8], some studies reported that statin therapy had no effect on insulin sensitivity [9–11], and others indicated a beneficial effect on glucose metabolism [12, 13]. The mechanisms by which statins may affect glycemic control have been under discussion [14], but not clear.

The target of the present study is to investigate the effect of simvastatin on FPG in STZ-induced diabetic rats. This research may provide an alternative to the problem of statin therapy. Toward this goal, we observed the different effect between simvastatin intervention rats and simvastatin treatment rats, especially on FPG levels of various periods. 

## 2. Methods

### 2.1. Materials

 Streptozotocin (Art. Number SO-130) was purchased from Sigma. Insulin ELISA test kits (Art. Number 10-1250-01) were purchased from Mercodia. Glucose, total cholesterol (TC), high-density lipoprotein cholesterol (HDL-C), low-density lipoprotein cholesterol (LDL-C), and triglyceride (TG) test kits were detected using automatic biochemistry analyzer. Simvastatin (Sino-American Shanghai Squibb Pharmaceuticals Ltd) was obtained from Squibb. Glucometer and glucose testing strips were productions of Roche (Switzerland). The standard diet was obtained from Animal Experimental Central, Shandong University (Jinan city, Shandong province); and the high-fat diet purchased from Beijing Ke' ao xieli company (Beijing city). The high-fat diet was purchased from standard diet (SD) consists of 6% fat, 64% carbohydrate and 23% protein, and high-fat diet (HFD) consists of 25% fat, 48% carbohydrate, and 20% protein.

### 2.2. Experimental Animals

Forty male Wistar rats, weighing 180–200 g, were obtained from Animal Experimental Central, Shandong University (Jinan city, Shandong province, China). Guides for the care and use of laboratory animals were approved by the Local Ethics Committee at the Medical College of Shandong University. Rats were housed in wire-floored cages under a 12 h light-dark cycle for at least 7 days prior to treatment and were fed with standard laboratory chow and tap water ad libitum. The room temperature was kept at 22 ± 2°C. All stressful conditions were avoided. Rats were fasted overnight prior to the study and housed in mesh-bottomed cages to minimize coprophagia. Except for the last hour, water was supplied ad libitum.

### 2.3. Experimental Induction of Diabetes

Food was withdrawn from 12 to 14 hours before the experiment, and diabetes was induced by a single intraperitoneal injection of a prepared solution of STZ (35 mg/kg) in 0.1 mol/L citrate buffer (pH 4.5). The dosing volume was 1 mL/kg. Successful induction of diabetes was confirmed by measuring the FPG concentration in rats 24 h after injection of STZ. The fasting blood glucose level above 11.1 mmol/L was considered diabetic and included in the present study.

### 2.4. Experimental Design

Wistar rats were fed with standard diet for one week, and then the rats were randomly divided into four groups, with ten rats in each group as follows: group A: normal control rats, Group B: diabetic model rats, Group C: simvastatin intervention rats, and Group D: simvastatin treatment rats. The normal control rats (*n* = 10) were fed with standard diet, receiving vehicle solution (citrate buffer, 1 mL/kg/day); Group B, Group C, and Group D rats (*n* = 24) were injected with STZ (35 mg/kg bodyweight, i.p.) after high-fat diet for 4 weeks. Successful induction of diabetes was confirmed by measuring the FPG concentration in rats 24 h after injection of STZ. The fasting blood glucose level above 11.1 mmol/L was considered diabetic rats. The simvastatin intervention rats received simvastatin (10 mg/kg/day) from the whole experiment and continued for 12 weeks, and simvastatin treatment rats were treated with simvastatin after successful induction of diabetes and continued for 8 weeks.

 The following parameters were assayed in each of the study groups during variance period: daily fluid and food consumption, weekly body weight, and blood glucose concentration. Food consumption was determined by subtracting leftovers from the diet provided to rats at 2-day intervals. 

The levels of fasting blood glucose, total cholesterol (TC), high-density lipoprotein cholesterol (HDL-C), low-density lipoprotein cholesterol (LDL-C), and triglyceride (TG) were assayed and compared with each group.

### 2.5. Statistical Analysis

The statistical analyses were conducted using SPSS18.0 software. Data are expressed as the mean value ± standard deviation. All groups of animals were studied in parallel. Comparisons among multiple groups were achieved via one-way ANOVA. Student's *t*-test was applied to compare probabilities of data between two groups. Values were considered significant when *P* < 0.05.

## 3. Results

### 3.1. Fluid and Food Intake and Bodyweight in Different Experimental Groups

Table 1 displays significant differences in fluid and food intakes and bodyweight change between normal control and diabetic model rats. Compared with normal control rats, there is a significantly increased fluid and food intakes and decreased bodyweight in diabetic rats. Intervention with simvastatin rats tended to increase the bodyweight to that seen in diabetic rats, and the effect was less pronounced in the group of rats treated with simvastatin.

### 3.2. Blood Glucose and Plasma Lipid Profile in Control and Diabetic Rats Are Given in Table 2

Table 2 shows that, in diabetic rats, there was a significant increase (*P* < 0.05) in TC and TG levels, and significant decrease in HDL-C, and the tendency of TC, TG, and HDL-C levels was even worse at the end of the experiment. Compared to untreated diabetic rats, the levels of TC and TG significantly decreased (*P* < 0.05) and the levels of HDL-C increased (*P* < 0.05) in simvastatin intervention rats. Compared to untreated diabetic rats the TC, TG, and HDL-C levels had no significant change in simvastatin treatment rats at 4 weeks, while there was a significant increase (*P* < 0.05) in TC and TG levels, and significant decrease in HDL-C at 12 weeks. 

### 3.3. The Different Effect between Simvastatin Intervention and Treatment Rats on FPG Is Given in Figure 1

**Figure 1:**
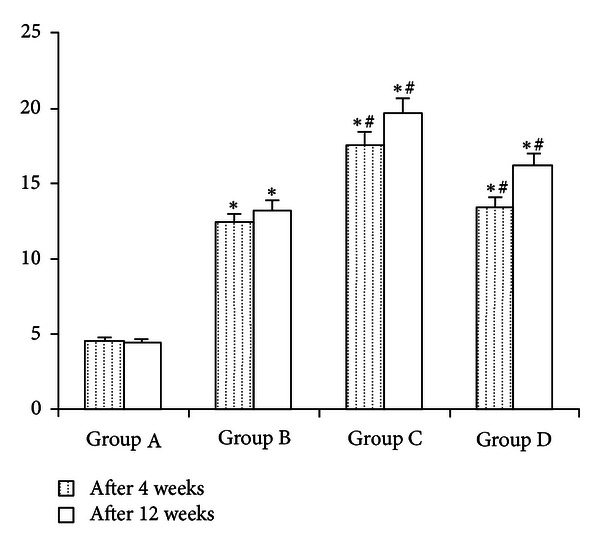
The different effect on FPG in each group of various periods. A: normal control group; B: diabetes model group; C: simvastatin intervention group; D: Simvastatin treatment group. *P < 0.05 compared with the control group. ^#^ *P* < 0.05 compared with the untreated diabetic group.

As summarized in Table 2 and Figure 1, at the end of 4 weeks and 12 weeks, the fasting blood glucose levels were significantly higher in diabetic animals when compared with control rat values, which is indicating that there was a successful induction of diabetic model rats. Compared with diabetic rats, at the end of 4 weeks, treatment of diabetic rats with simvastatin slightly increased FPG (*P* > 0.05) simvastatin intervention rats were significantly increased FPG (*P* < 0.05) and easily induced diabetic rats. At the end of 12 weeks, simvastatin intervention rats were still higher at the level of FPG, while the FPG levels were slightly decreased compared with the end of 4 weeks. At the end of 12 weeks, the simvastatin treatment rats had a significantly higher FPG levels compared with the diabetic rats (*P* < 0.05).

## 4. Discussion

 Statins can enhance the expression of low-density lipoprotein (LDL) receptors in the liver and consequently lower the levels of blood LDL cholesterol through inhibiting cholesterol synthesis in the liver [15]. Stains, including both water-soluble and lipid-soluble statins, have been shown to protect against the injury in cardiovascular and renal diseases [16, 17]. Besides their well-established role in lowering cholesterol levels, recent research has demonstrated that statins might have relevant effect on glucose metabolism in animal models and in humans. However, several studies on the effect of statin on glucose metabolism are controversial. Some [18–20] have shown that simvastatin had a benefit effect on insulin resistance; some [21, 22] have reported no benefit on insulin action, and others [23–25] have identified a deterioration in glucose homoeostasis. These mechanisms may be caused by the different property of statins. Water-soluble statins, such as rosuvastatain, are hepatocyte specific and are not probably taken up by pancreatic cells and adipocytes. Lipid-soluble statins, such as simvastatin, enter extrahepatic cells easily and may suppress isoprenoid protein synthesis, consequently attenuating the action of insulin levels. The present study is to investigate the effect of simvastatin on glucose metabolism from the prediabetic rats models. Toward this goal, we observed the different effect between simvastatin intervention rats and simvastatin treatment rats, especially on FPG levels of various periods. 

This study shows that the rats with simvastatin intervention at a dose of 10 mg/kg have increased the levels of FPG, and consequently the diabetic model rats were easily induced. Similarly, we observed that FPG was slightly increased in diabetic rats after eight weeks with simvastatin treatment. In addition to the classic function of lowering the cholesterol levels, our study demonstrated that statin could also impact the glucose homeostasis on STZ-induced diabetic rats. Despite the levels of TC and TG decreased and total cholesterol/HDL ratio increased compared with control group, both intervention and treatment with simvastatin rats did not improve the glucose homeostasis, especially on FPG. These results suggest that the rats with simvastatin intervention were more easily induced, the diabetic model rats and the diabetic rats, those treated with simvastatin could also increase the levels of FPG. Therefore simvastatin could not decrease the FPG levels and improve the insulin sensitivity. What is more, simvastatin therapy is associated with a slightly increased risk of diabetes.

In this paper, we demonstrated the different effect on FPG between intervention and treatment with simvastatin rats. The FPG increased greater in simvastatin intervention rats than simvastatin treatment rats. Importantly, the simvastatin intervention rats were more easily induced to be diabetic rats and had a higher FPG compared with simvastatin treatment group. Besides, the results indicated that simvastatin intervention rats were more inclined to have an adverse effect on glucose homeostasis, especially on FPG, compared with simvastatin treatment rats. The biological significance of elevated FPG identified in simvastatin intervention group remains unknown, but some studies [26] would suggest that the abnormality in FPG may translate into the clinical syndrome of diabetes with a rise in glycosylated hemoglobin (HbA1c). In addition, it has provided that there is a graded relationship between FPG and the extent of coronary artery disease severity [27]. Current estimates indicate that simvastatin intervention rats, with prediabetic status, may easily develop diabetic rats induced by STZ. It is noteworthy that, in our current study, the mean FPG value in simvastatin treatment rats increased which is in the impaired fasting glucose range. The worsening of FPG in simvastatin intervention was even more pronounced.

The precise mechanisms by which statins may exert any influence on glucose metabolism are unclear. Statins have the potential to alter glycemic control by decreasing various metabolites such as isoprenoid, farnesyl pyrophosphate, geranylgeranyl pyrophosphate, and ubiquinone (CoQ10), all of which are dependent on mevalonic acid production. Isoprenoid in particular enhances glucose uptake via GLUT-4 in adipocytes [28]. Reduction in CoQ10 may result in delayed ATP production in pancreatic beta cells and thereby impair insulin release [29]. The mechanisms may have the relationship with the property of statins. Simvastatin has been shown to inhibit glucose-induced increase in intracellular calcium in pancreatic beta cells leading to the inhibition of insulin secretion in a dose-dependent manner [30]. Thus, this study shows that simvastatin intervention that effectively increased FPG may promote the development of type 2 diabetes mellitus. The research may provide an alternative to the problem of statin therapy.

The limitations of the research showed that our diabetic rats were followed up for 8 weeks in this experimental study this is an obviously short period considering the natural history of diabetes mellitus. We do not know whether this association would continue, worse, or get better through a long period of followup. The biological importance of elevated FPG in simvastatin intervention and treatment rats identified in this study remains unknown, but data from the JUPITER trial [26] would suggest that the elevated FPG may translate into the clinical syndrome of diabetes with a rise in HbA1C. Besides, it has been demonstrated that there is a graded relationship between FPG and the levels of coronary artery disease severity [31]. The present estimates indicate that most individuals with prediabetic status may eventually develop clinical diabetes mellitus. 

## 5. Conclusion

Our observational study demonstrated that statin therapy is associated with a rise of FPG levels over a mean period of 12 weeks in simvastatin intervention rats. Considering the prevalence of statin usage in current clinical practice, this significant observation merits further investigation. Clinicians should realize this potential adverse association of statin use on FPG, and careful monitoring is advised.

## Figures and Tables

**Table 1:** Administration of simvastatin in fluid and food intake and body weight of rats in the different experimental groups.

Groups	Fluid intake (mL/day)	Food intake (g/day)	Weight gain (g/day)
Normal control	24.00 ± 4.00	19.3 ± 0.2	5.20 ± 0.02
Diabetic	116.00 ± 5.00*	28.50 ± 0.40*	3.10 ± 0.03*
Simvastatin intervention	40.00 ± 2.00^∗#^	24.50 ± 0.30*	3.80 ± 0.05^∗#^
Simvastatin treatment	56.00 ± 3.00^∗#^	25.40 ± 0.20*	3.50 ± 0.03*

Data are the mean ± SD for ten animals in each group.

*P < 0.05 compared with the control group.

^#^ *P* < 0.05 compared with the untreated diabetic group.

**Table 2:** Effect of intervention and treatment with simvastatin on fasting plasma glucose, serum total cholesterol, HDL-C, and triglycerides.

Groups	FPG (mmol/L)	Total cholesterol (mmol/L)	HDL-C (mmol/L)	Triglycerides (mmol/L)
Normal control				
Baseline	4.5 ± 0.8	2.0 ± 0.2	0.85 ± 0.02	1.3 ± 0.1
After 4 weeks	4.6 ± 0.6	2.1 ± 0.1	0.87 ± 0.01	1.2 ± 0.2
After 12 weeks	4.4 ± 0.3	2.2 ± 0.3	0.92 ± 0.03	1.1 ± 0.2
Diabetic				
Baseline	4.8 ± 0.6	1.9 ± 0.1	0.75 ± 0.01	1.2 ± 0.1
After 4 weeks	12.4 ± 1.2*	3.7 ± 0.8*	0.59 ± 0.02*	2.6 ± 0.3*
After 12 weeks	13.2 ± 0.9*	4.0 ± 0.5*	0.35 ± 0.01*	3.4 ± 0.2*
Simvastatin intervention				
Baseline	4.6 ± 0.1	2.1 ± 0.2	0.83 ± 0.02	1.4 ± 0.1
After 4 weeks	17.5 ± 0.9^∗#^	2.2 ± 0.3^ #^	1.34 ± 0.04^∗#^	1.2 ± 0.3*
After 12 weeks	15.1 ± 0.2^∗#^	2.0 ± 0.1^#^	1.56 ± 0.02^∗#^	1.3 ± 0.2*
Simvastatin treatment				
Baseline	4.2 ± 0.5	2.0 ± 0.1	0.87 ± 0.03	1.3 ± 0.1
After 4 weeks	13.4 ± 1.5*	3.2 ± 0.1^∗#^	0.62 ± 0.02^#^	2.2 ± 0.2^#^
After 12 weeks	16.2 ± 1.0^∗#^	2.1 ± 0.3^∗#^	1.49 ± 0.05^#^	1.5 ± 0.3^#^

Data are the mean ± SD for ten animals in each group.

*P < 0.05 compared with the control group.

^#^ *P* < 0.05 compared with the untreated diabetic group.
